# Assessing the Contribution of Self-Monitoring Through a Commercial Weight Loss App: Mediation and Predictive Modeling Study

**DOI:** 10.2196/18741

**Published:** 2021-07-14

**Authors:** Gregory Farage, Courtney Simmons, Mehmet Kocak, Robert C Klesges, G Wayne Talcott, Phyllis Richey, Marion Hare, Karen C Johnson, Saunak Sen, Rebecca Krukowski

**Affiliations:** 1 Department of Preventive Medicine College of Medicine University of Tennessee Health Science Center Memphis, TN United States; 2 Center for Addiction Prevention Research Department of Public Health Sciences University of Virginia Charlottesville, VA United States

**Keywords:** weight loss, self-monitoring, obesity, apps, behavioral intervention

## Abstract

**Background:**

Electronic self-monitoring technology has the potential to provide unique insights into important behaviors for inducing weight loss.

**Objective:**

The aim of this study is to investigate the effects of electronic self-monitoring behavior (using the commercial *Lose It!* app) and weight loss interventions (with differing amounts of counselor feedback and support) on 4- and 12-month weight loss.

**Methods:**

In this secondary analysis of the Fit Blue study, we compared the results of two interventions of a randomized controlled trial. Counselor-initiated participants received consistent support from the interventionists, and self-paced participants received assistance upon request. The participants (N=191), who were active duty military personnel, were encouraged to self-monitor their diet and exercise with the Lose It! app or website. We examined the associations between intervention assignment and self-monitoring behaviors. We conducted a mediation analysis of the intervention assignment for weight loss through multiple mediators—app use (calculated from the first principal component [PC] of electronically collected variables), number of weigh-ins, and 4-month weight change. We used linear regression to predict weight loss at 4 and 12 months, and the accuracy was measured using cross-validation.

**Results:**

On average, the counselor-initiated–treatment participants used the app more frequently than the self-paced–treatment participants. The first PC represented app use frequencies, the second represented calories recorded, and the third represented reported exercise frequency and exercise caloric expenditure. We found that 4-month weight loss was partially mediated through app use (ie, the first PC; 60.3%) and the number of weigh-ins (55.8%). However, the 12-month weight loss was almost fully mediated by 4-month weight loss (94.8%). Linear regression using app data from the first 8 weeks, the number of self–weigh-ins at 8 weeks, and baseline data explained approximately 30% of the variance in 4-month weight loss. App use frequency (first PC; *P*=.001), self-monitored caloric intake (second PC; *P*=.001), and the frequency of self-weighing at 8 weeks (*P*=.008) were important predictors of 4-month weight loss. Predictions for 12-month weight with the same variables produced an *R*^2^ value of 5%; only the number of self–weigh-ins was a significant predictor of 12-month weight loss. The *R*^2^ value using 4-month weight loss as a predictor was 31%. Self-reported exercise did not contribute to either model (4 months: *P*=.77; 12 months: *P*=.15).

**Conclusions:**

We found that app use and daily reported caloric intake had a substantial impact on weight loss prediction at 4 months. Our analysis did not find evidence of an association between participant self-monitoring exercise information and weight loss. As 12-month weight loss was completely mediated by 4-month weight loss, intervention targets should focus on promoting early and frequent dietary intake self-monitoring and self-weighing to promote early weight loss, which leads to long-term success.

**Trial Registration:**

ClinicalTrials.gov NCT02063178; https://clinicaltrials.gov/ct2/show/NCT02063178

## Introduction

### Background

Consistent dietary and physical activity self-monitoring is an important component of successful weight loss in both traditional in-person behavioral weight loss programs [1,2] and technology-based programs [2-5]. Technology may also increase self-monitoring adherence [2,6-8]. Perhaps because technology-based dietary and physical activity self-monitoring requires minimal effort [9], commercial technology-based dietary and physical activity monitoring programs have grown in quantity and popularity with apps such as *Lose It!*, which has reported more than 30 million downloads to date, and MyFitnessPal, which had 225 million users in 2018 [10,11].

Self-weighing is another form of self-monitoring that facilitates weight loss [12-14], perhaps because of the behavioral changes in diet or exercise that occur as participants become more aware of their weight trajectories [15,16]. Until recently, many studies involving self-weighing relied on questionnaires in which participants could specify their weighing frequency [12]; now, it is possible to directly measure adherence to self-weighing through smart scales that record a participant’s weight and self-weighing habits and transmit this information through wireless cellular technology.

Early performance (ie, self-monitoring adherence and weight loss) in a weight loss program is an indicator of long-term weight loss [17-20]. Tsai et al [17] showed that more detailed food records before randomization led to greater weight loss at 1 year. Similarly, Krukowski et al [19] found that early dietary and physical activity self-monitoring is a predictor of weight loss success. In a study by Unick et al [20], weight change at 2 months was predictive of weight change 8 years later. Early identification of participants who are not self-monitoring and who may be at most risk of not losing weight gives clinicians and researchers the opportunity to target those falling behind with additional resources.

### Objectives

The first aim of our investigation was to increase our understanding of self-monitoring behaviors that lead to successful weight loss by using a commercial weight loss app, Lose It!. The second aim was to study if app use predicted weight loss at 4 and 12 months using participant data at an early stage (eg, 4 weeks, 8 weeks, and 4 months) to better identify individuals who might need early attention. As the app use data were composed of many interrelated variables, we summarized the variables using principal component analysis (PCA), which also addresses multicollinearity. We implemented a mediation analysis under the counterfactual framework to understand the mechanism of action of intervention assignment on weight loss at 4 and 12 months. We performed linear regression models with variables from the PCA analysis from the first 4 and 8 weeks of this study to predict weight loss at 4 and 12 months in the participants.

The prediction analysis allowed us to statistically model the relationship between the weight loss variables (at 4 and 12 months) and our set of independent predictors. It revealed the relationships among the variables; however, it did not indicate whether these relationships are causal. The complementary mediation analysis permitted us to make causal inferences about the effect of treatment assignment on weight loss through the mediators.

## Methods

### Study Design

This is a secondary analysis of the Fit Blue study, which was adapted from the Look AHEAD (Action for Health in Diabetes) Intensive Lifestyle Intervention [21-23] for a military lifestyle. The participants were randomly assigned to 1 of 2 treatment groups using a randomized block design with block size 4 [24]. The 2 treatment groups, counselor-initiated treatment and self-paced treatment, differed in the amount of self-initiation required to receive treatment. The counselor-initiated group received 28 phone calls over 12 months with counselors, regular feedback through email on their self-monitored entries on the same schedule as their phone sessions, 28 lesson materials, meal replacements, individualized detailed exercise and meal plans, and access to materials such as food scales, exercise videos, resource books, and cookbooks. They were also encouraged to participate in four challenges to boost their motivation. The self-paced participants could receive the same number of phone sessions and email feedback as the counselor-initiated participants upon request. The self-paced participants also had access to lesson materials and exercise and meal plans, although they had to initiate the request for assistance. Further details about the study design, meal replacements, and study website can be found elsewhere [24]. The main outcome as well as the treatment engagement outcomes have been previously published [25].

The behavioral change goals were standard across the two conditions. All participants were asked to record their daily dietary intake and physical activity for 12 months on the Lose It! app or website. Lose It! premium accounts were created for all participants. They were encouraged to track their diet, caloric intake, and exercise using the app. The Lose It! app gave the participants access to a database with more than 7 million foods and corresponding nutritional information [26]. They were encouraged to lose 10% or more of their initial body weight at a rate of 1-2 pounds per week. Participants with a starting weight of less than 79 kg, between 79 kg and 97.5 kg, and more than 97.5 kg were given daily calorie goals of 1200-1300 kcal, 1500-1600 kcal, and 1800-1900 kcal, respectively. All participants were given a daily goal of consuming no more than 30% of calories from fat. In addition, the participants received a personalized exercise plan based on their self-reported physical activity at the baseline visit. They were asked to gradually increase aerobic exercise from their current level reported at baseline until reaching 225-250 minutes weekly, at which point they were to maintain this amount of exercise. A BodyTrace e-scale was provided to each participant, and they were requested to self-weigh daily. In addition, they were asked to attend two in-person follow-up data collection visits at 4 and 12 months.

### Participants

A total of 248 active duty military personnel at Joint Base San Antonio (previously named Lackland Air Force Base) in San Antonio, Texas, participated in the Fit Blue study [24]. To be eligible to participate, participants had to meet the following criteria: aged 18 years or older, BMI of 25 kg/m^2^ or more, access to a computer and email, and clearance from a health care provider. In addition, 1 week of monitoring dietary intake and physical activity on Lose It! was required for eligibility [24,27].

Participant recruitment began in December 2013 and ended in March 2016. The Fit Blue study was approved by the institutional review board of the Wilford Hall Ambulatory Surgical Center and acknowledged by the institutional review board of the University of Tennessee Health Science Center. The study approval was maintained over the course of the study, and a data and safety monitoring officer reviewed the accumulated data.

### Measures

#### Physical Measurements

The primary outcome was weight change (in kilograms) measured on a calibrated scale (Tanita BWB-800S) in street clothes and without shoes. Height (in centimeters) was measured without shoes using a wall-mounted stadiometer. These measures were recorded at the baseline, 4-month weigh-in, and 12-month data collection visits. Our outcome variable, that is, weight loss, was modeled as the log ratio of the final weight at 4 and 12 months over the baseline weight. We log-transformed the weight change to stabilize variance and address the skewness of the distribution. As we are mostly interested in the participant’s achievement, the weight loss ratio was classified as success (≥5% loss), some loss (2.3%-5%), or no loss (<2.3%). A 5% weight loss represents a benchmark at which point clinical benefits are observed [28]. Weight loss of less than 2.3% (or approximately 2.3 kg) has been used in previous research to denote weight stability [29].

#### Weight Self-Monitoring Behaviors

The participants were asked to monitor their weight daily using the BodyTrace e-scale. The time-stamped weights were transmitted to the study team over cellular technology, and they were also uploaded to each participant’s personalized website to view their progress. The self-weighing variable represents the number of days the participants weighed themselves using the BodyTrace e-scale. We included only the plausible values filtered by the True Profile Finder algorithm [30].

#### Sociodemographic Characteristics

Age, education level, race, and gender were collected through a baseline questionnaire.

#### Dietary and Physical Activity Monitoring Behaviors

The participants recorded their daily consumption (ie, food and beverage items along with their calories) and exercise (ie, type and duration) using Lose It!. The participants were able to log food items for meals, snacks, and beverages, as well as exercise type and volume. They could also use the app to log their weight, which was counted as logging but not as the self-weighing variable. A total of 9 logging-specific measures were calculated to quantify logging behavior, and six caloric measures were estimated to assess caloric intake. We measured the average of the total number of days each week that participants logged at least 1 entry for food, beverage, exercise, or weight.

**Figure 1:**
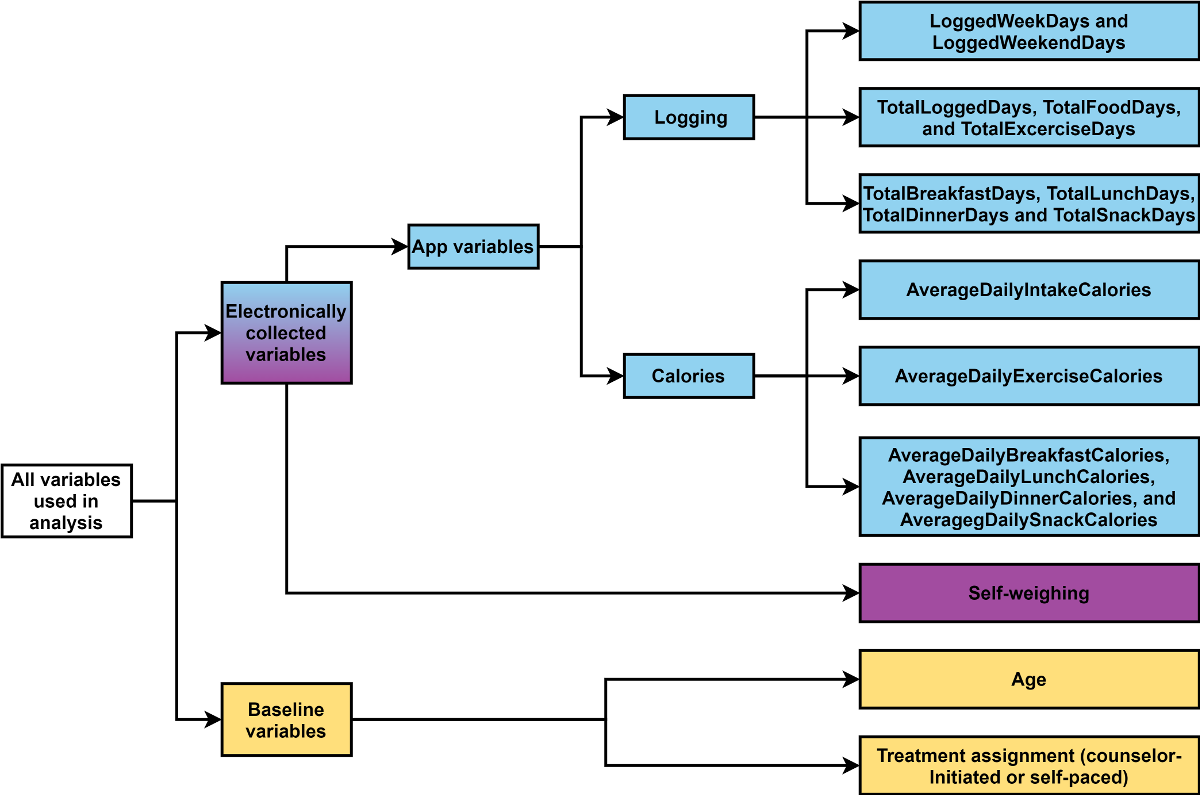
Diagram of variables used in prediction models and mediation analysis.

The independent variables included in the analysis (Figure 1) were categorized into two groups: baseline variables and electronically collected variables. The baseline variables consisted of age and treatment assignment. The electronically collected variables consisted of the frequency of 16 self-weighing and Lose It! app variables (Textbox 1). Each of the measures was calculated for a specific time period (ie, the first 4 weeks, the first 8 weeks, 4 months, and 12 months) depending on the analysis. The time periods used are mentioned in the analysis sections.

**Description of the electronically collected variables.**

**Logging measures**

- *LoggedWeekDays* and *LoggedWeekendDays* are the number of weekdays and weekend days, respectively, that include at least 1 entry for food, beverage, exercise, or weight.
- *TotalLoggedDays *is the total number of days with at least 1 entry for food, beverage, exercise, or weight.
- *TotalFoodDays* is the total number of days with at least 1 food or beverage entry.
- *TotalExerciseDays* is the total number of days with at least 1 entry for exercise.
- *TotalBreakfastDays*, *TotalLunchDays*, *TotalDinnerDays*, and *TotalSnackDays* are the total number of days with at least 1 entry for breakfast, lunch, dinner, and snack, respectively.

**Caloric measures**

- *AverageDailyIntakeCalories* is the average daily intake of calories from food over the total number of days with at least 1 food entry. The entries for daily calories that exceeded an upper bound (ie, 4000 kcal for women and 5000 kcal for men) and lower bound (ie, 600 kcal for women and 800 kcal for men) were excluded for implausibility [31].
- *AverageDailyExerciseCalories* is the average daily calories burned through exercise over the total number of days with at least 1 entry for exercise.
- *AverageDailyBreakfastCalories*, *AverageDailyLunchCalories*, *AverageDailyDinnerCalories*, and *AverageDailySnackCalories* are the average daily caloric intake values over the total number of days with at least 1 entry for breakfast, lunch, dinner, and snack, respectively.

### Statistical Analysis

#### Overview

The analysis was conducted using R version 4.0.4 [32]. We summarized the data and created graphics for descriptive analysis using the tidyverse package (version 1.3.0) [33]. Descriptive statistics were calculated to examine changes in logging frequency over the course of the study. We considered all sociodemographic variables collected for analysis and included them only if they were associated with a *P* value of .20 or less with the weight loss outcome. Using this criterion, only age was included in the final analysis.

#### Loss to Follow-up

As this is a longitudinal study, not all individuals’ weights were observed at the 4- and 12-month visits. Our main analysis included only individuals with complete weight data. However, we also investigated the sensitivity of the effect of the missing data using three different analyses. In the last observation carried forward analysis, we assumed that there was no change in the participants’ weight after missing a visit. With a more conservative approach, the baseline observation carried forward analysis assumption is that the participants who missed a visit returned to their baseline weight. Neither method showed a substantial difference in the outcome. The results are detailed in the supplemental material available on a GitHub repository [34].

#### Principal Component Analysis

We performed a PCA on the Lose It! app variables to reduce the number of variables considered and still capture the useful information in these variables. The PCA also had the additional advantage of addressing multicollinearity in the variables [35]. Each principal component (PC) was a linear combination of the original variables and was uncorrelated with the other PCs. The PC that captured the largest variance was called the first PC, and the PC that captured the second largest variance was called the second PC, and so on [36,37]. In our case, we had 15 electronically collected variables, of which some were highly correlated with each other. The PCA reduced the number of uncorrelated variables to a much smaller number. We used the packages FactoMineR (version 2.4) [38] and factoextra (version 1.0.7) [39] to obtain the PCs and the new uncorrelated variables. We used a scree plot to determine the number of PCs to include in the analysis. The PCs that jointly explained 70% of the variance in the data were included in the analysis. Depending on the analysis of interest, PCA was applied to the variables measured over a specific period. Textbox 2 specifies these periods and the data used in the corresponding analyses.

**Time period for the electronically collected variables according to the analysis.**

**Mediation for 4-month weight loss**

- Baseline to 4 months

**Mediation for 12-month weight loss**

- Baseline to 12 months

**Prediction of the 4-month weight loss**

- Baseline to 4 weeks or baseline to 8 weeks data

**Prediction of the 12-month weight loss**

- Baseline to 4 weeks or baseline to 8 weeks data

To identify the most important variables that explain the variations in our data, we studied the quality of the representation and the contribution of the variables. The quality of representation of a variable is determined by the square of the correlation coefficient between a variable and a PC. The contribution of a variable for a given PC is estimated by the ratio of its squared correlation coefficient over the sum of all squared correlation coefficients between each variable and the given component [39].

#### Mediation

**Figure 2:**
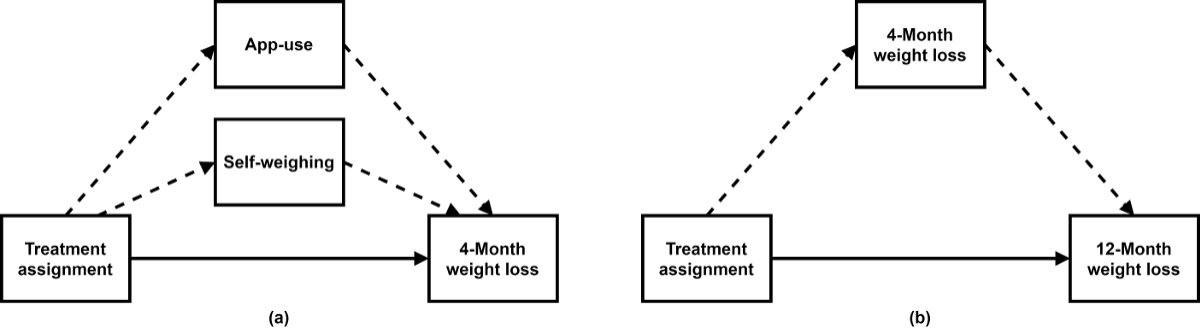
Diagram representing our causal model for treatment assignment, mediators, and outcomes. Arrows show the direction of causation. Dashed arrows represent mediated effects, and solid arrows represent direct effects. a: Causal model for the 4-month weight loss outcome. b: Causal model for the 12-month weight loss outcome.

We conducted a causal mediation analysis to understand whether, and how much of, the intervention assignment effect on weight loss operates through intermediate variables such as *app use* and *self-weighing* frequency. Figure 2 depicts the hypothesized causal framework underlying our mediation analysis. The analysis was based on a counterfactual framework using a linear regression analytic approach [40]. Simply put, this framework partitions the total effect (TE) of the intervention assignment into the sum of the average causal mediation effect (ACME) and average direct effect. It can be shown that this approach can be used with both linear and nonlinear models and is equivalent to the traditional approach of MacKinnon et al [41] when some conditions hold [42]. In the counterfactual framework, we need to assume two statements to make valid inferences about the causal mediation analysis. This assumption is known as the sequential ignorability assumption. First, the treatment variable is statistically independent of the outcome and mediator variable; second, the mediator is independent of the outcome, given the observed exposure and pretreatment confounders. The counterfactual framework introduced by Imai et al [42] allows us to assess the robustness of our causal conclusions with respect to the violation of the sequential ignorability assumption. To establish if any association existed between the intervention assignment variable and the outcome of weight loss (at 4 and 12 months) and each mediator, we used the two-tailed *t* statistic. Significance was established using 10,000 permutations. In the causal mediation analysis [42], if the treatment assignment has no effect on the mediator, then the causal mediation effect is zero. The randomized exposure variable (ie, intervention assignment) has two states: self-paced or counselor-initiated. We conducted two mediation analyses for two outcomes: the 4-month weight loss and the 12-month weight loss. In the first mediation analysis, we considered two mediators of the 4-month weight loss: app use (calculated from the first PC of the electronically collected variables) and the frequency of self-weighing. In the second mediation analysis, we considered the mediation of the 12-month weight loss by the 4-month weight loss itself (Figure 2).

The mediation analysis included all the data accumulated during the entire study, up to 4 months and 12 months, according to the period of interest. In all models for mediation, we included baseline covariates (age). To estimate the ACME (ie, indirect effect) and the average direct effect from the model-based causal mediation analysis, we used the package mediation (version 4.5.0) [43]. The uncertainty estimates were computed using a nonparametric bootstrap [43,44] with 10,000 simulations. The main motivation is not only to investigate whether a mediation exists, but also to determine if it is partially or fully mediated. It is fully mediated only if the direct effect is zero and partially mediated otherwise. As we had multiple mediators, we were interested in identifying the mediator that conveyed the greatest effect on weight loss [40,45-47].

#### Weight Loss Predictions Through Linear Regression

We used the mediation results to guide our variable selection process for the prediction. Five models were considered for 4-month weight loss, and six models were considered for 12-month weight loss. These models used a combination of baseline and electronically collected variables (ie, frequency of self-weighing and app variables summarized by PCA) as predictor variables. We predicted 4-month weight loss and 12-month weight loss using three different sets of predictor variables: (1) only baseline variables (ie, age and treatment assignment), (2) only electronically collected variables from the first 4 weeks, and (3) baseline and electronically collected variables from the first 4 weeks combined. The latter two models were repeated using electronically collected variables from the first 8 weeks. Finally, we considered one more model for 12-month weight loss using 4-month weight loss as the only predictor variable.

We used five-fold cross-validation to evaluate the linear regression models. The PCA was conducted using four out of five folds, including samples with a response value and incomplete samples without response values [48]. The resulting PCs were used to create a linear regression model. The model predicted log weight loss for the fifth fold composed of complete samples. This process was repeated until all participants’ weight loss was predicted. We categorized actual and predicted weight loss values as successful weight loss, some loss, or no loss. Prediction accuracy was evaluated using *R*^2^, the Spearman rank correlation coefficient (ρ), and the multi-class area under the receiver operating characteristic curve (AUC) value. The folds and cross-validation were performed using the caret package (version 6.0-80).

## Results

### Overview

**Figure 3:**
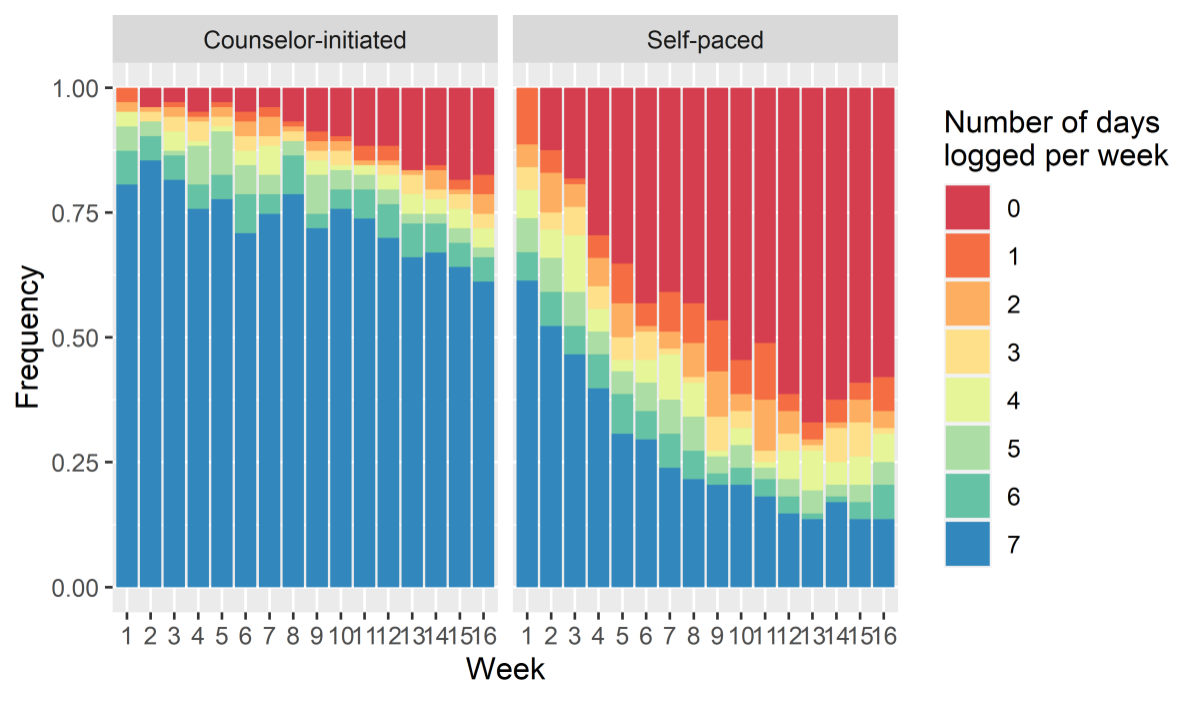
Number of days per week with at least one logging (by week and treatment groups) during the first 4 months.

Owing to technical problems when downloading the data for 57 of the participants, 191 of the 248 randomized subjects were included in the analysis (including 103 in the counselor-initiated treatment and 88 in the self-paced treatment). Approximately 80.6% (154/191) of the participants attended the 4-month data collection visit, and approximately 80.1% (153/191) participated in the 12-month data collection visit. The study demographics are summarized in Table 1. At 4 months, the counselor-initiated treatment lost an average of 3.7 kg (SD 3.6), and the self-paced treatment lost 0.6 kg (SD 3.1). At 12 months, the counselor-initiated treatment lost 2.4 kg (SD 5.0) on average, and the self-paced treatment gained 0.2 kg (SD 5.1). Figure 3 shows the differences in the number of days with at least 1 item logged per week between the treatment groups. The counselor-initiated treatment ’s average number of days with at least 1 item logged (ie, food, beverage, exercise, or weight entries) decreased from 6.48 days per week (week 1) to 5.03 days per week (week 16) over the first 4 months of the study and dropped to 0.70 days per week (week 52) by 12 months. The logging instances for the self-paced treatment decreased from 5.55 days per week (week 1) to 1.99 days per week (week 16) at 4 months and finally to 0.01 days per week (week 52) days by 12 months.

**Table 1:** Demographics of counselor-initiated and self-paced participants (N=191).

Characteristics			Counselor-initiated participants (n=103)		Self-paced participants (n=88)		Total (N=191)		*P* value	
**Gender, n (%)**										.99
	Female	53 (51.5)		46 (52.3)		99 (51.8)				
	Male	50 (48.5)		42 (47.7)		92 (48.2)				
**Race, n (%)**										.66
	Black or African American	22 (21.4)		16 (18.2)		38 (19.9)				
	White	65 (63.1)		61 (69.3)		126 (65.9)				
	Other	16 (15.5)		11 (12.5)		27 (14.1)				
**Education, n (%)**										.55
	Less than college	46 (44.7)		44 (50)		90 (47.1)				
	College or above	57 (55.3)		44 (50)		101 (52.9)				
**BMI (kg/m^2^), n (%)**										.37
	Overweight (BMI 25-29.9)	51 (49.5)		37 (42)		88 (46.1)				
	Obese (BMI≥30)	52 (50.5)		51 (58)		103 (53.9)				
Age (years), mean (SD)			35.5 (8.2)		33.9 (6.8)		34.8 (7.6)		.14	

The app variables were summarized using PCA with data from the first 4 and 8 weeks. A total of 72.8% of the variation in the 8-week data was captured in the first two components.

**Figure 4:**
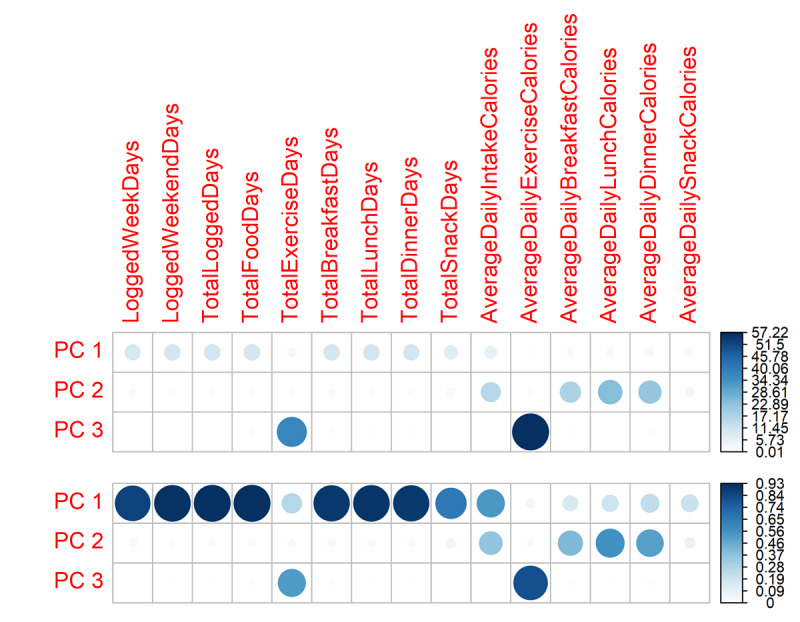
The contribution of variable results (top) for the first 3 PCs from the 8-week PC analysis runs on a scale of 0 to 100. The darker and larger a circle, the more it contributed to a PC. The quality of the representation of variable results (bottom) are on a scale of 0 to 1. The darker and larger a circle, the more it is represented by a PC compared with other PCs. PC: principal component.

Although the PCs are linear combinations of the original variables, they have some interpretive value, that is, we still preserved some cohesive interpretations of the three first PCs. Figure 4 shows that PC_1_ mainly comprises app variables describing the frequency with which the participants used the app; therefore, we labeled PC_1_ *app-use,* and it explains 57.5% of the variance. PC_2_ mostly describes daily caloric intake that was self-monitored by the participants, and it explains 15.3% of the variance. We labeled PC_2_ *app-calories*. The inclusion of PC_3_ explains 9.4% of the variance (82.2% total), and because this component represents the participants’ exercise self-monitoring, it is labeled *app-exercise*.

### Mediation

We estimated the significance of the relationship between the intervention assignment variable (self-paced or counselor-initiated) and weight loss for two different periods (ie, baseline to 4 months and baseline to 12 months). There was a significant relationship between the treatment assignment variable (self-paced or counselor-initiated) and weight loss at 4 months (*P*<.001) and 12-months (*P*=.002).

For the two periods of interest (4 months and 12 months), treatment assignment was significantly associated (*P*<.001) with each mediator: the *app-use* component (PC_1_), the *app-calories* component (PC_2_), self-weighing frequency, and 4-month weight loss. However, treatment assignment was not associated with the *app-exercise* component (PC_3_; *P*=.73). We considered only *app-use* (PC_1_) as a mediator because it explained more than 50% of the variance by itself in comparison with *app-calories* (PC_2_; <20%) and *app-exercise* (PC_3_; <10%), which explained a much smaller proportion of the variance.

During the first 4 months (Table 2), we found that the indirect intervention’s effect on weight loss transmitted through *app-use* (PC_1_) and self-weighing frequency roughly accounted for 60% and 55% of the TE, respectively. For weight loss at 12 months, the indirect effect (ACME: 0.030, 90% CI 0.0017-0.046) through the 4-month weight loss accounted for 94% of the TE. The TE denotes how much weight loss would change overall if treatment assignment was changed from the self-paced to the counselor-initiated intervention. Table S7 in Multimedia Appendix 1 [44] contains the results of the sensitivity analysis; more details are provided in the GitHub repository [34].

**Table 2:** The 4- and 12-month mediation estimates the total effect, average direct effect (ADE), average causal mediation effect (ACME), and proportion mediated effect.

Mediator			Total effect (90% CI)		ADE^a^ (90% CI)		ACME^b^ (90% CI)		Proportion mediated, %
**4-month mediation**									
	App-use (PC_1_^a^)	0.034 (0.023 to 0.045)		0.013 (0.002 to 0.025)		0.021 (0.012 to 0.029)		60.3	
	Self-weighing frequency	0.034 (0.023 to 0.045)		0.015 (0.004 to 0.026)		0.019 (0.012 to 0.027)		55.8	
**12-month mediation**									
	4-month weight loss	0.032 (0.015 to 0.048)		0.002 (−0.012 to 0.031)		0.03 (0.019 to 0.043)		94.8	

^a^PC_1_: principal component 1.

### Weight Loss Predictions Through Linear Regression

The cross-validated linear models built on baseline variables and electronically collected variables (ie, *app-use* [PC_1_], *app-calories* [PC_2_], *app-exercise* [PC_3_], and self-weighing frequency) were first compared for weight loss prediction accuracy using 4-week data. For 4-month weight loss, the 8-week model explained approximately 4% more variance than the model built on 4-week data (*R*^2^=0.30 and 0.26, respectively). For 12-month weight loss, *R*^2^ also slightly increased when 8-week data were used compared with using 4-week data (*R*^2^=0.08 and 0.06, respectively).

As results with 8-week data showed marginal improvement when compared with those using 4-week data, this study primarily focuses on analysis with 8-week data. The results from the 8-week model performance are shown in Table 3. More information on the 4-week results and scatterplots of the models can be found in Multimedia Appendix 1.

**Table 3:** Accuracy of 8-week models for predicting 4-month and 12-month weight losses.

Model	4-month prediction				12-month prediction		
	*R* ^2^	ρ	mAUC^a^	*R* ^2^		ρ	mAUC
Baseline variables (ie, age and treatment assignment)	0.16	0.41	0.65	0.06		0.25	0.58
App-use (PC_1_^b^)+app-calories (PC_2_^c^)+self-weighing frequency	0.31	0.58	0.74	0.07		0.29	0.57
Baseline variables+app-use (PC_1_)+app-calories (PC_2_)+self-weighing frequency	0.30	0.58	0.73	0.08		0.29	0.58
4-month weight loss	N/A^d^	N/A	N/A	0.31		0.52	0.66

^a^mAUC: multi-class area under the receiver operating characteristic curve.

^b^PC_1_: principal component 1.

^c^PC_2_: principal component 2.

^d^N/A: not applicable.

For 4-month predictions, cross-validation of only the baseline variables using a linear model resulted in modest *R*^2^ and ρ values (*R*^2^=0.16; ρ=0.41; AUC=0.65). Adding the electronically collected variables (ie, *app-use* [PC_1_], *app-calories* [PC_2_], and self-weighing frequency at 8 weeks) to the model explained approximately 15% more variance than the baseline variables alone (*R*^2^=0.30; ρ=0.58; AUC=0.73). The 12-month predictions were less successful than the 4-month predictions when considering a combination of baseline variables and electronically collected variables (Table 3). Using all analysis variables only accounted for 8% of the variance in the data (ρ=0.29; AUC=0.58) for the 12-month prediction. For both the 4-month and 12-month results, predictions based on only electronically collected variables tended to be slightly better than the predictions from the models that included baseline variables. This suggests that treatment assignment and age do not improve out-of-sample prediction when technological variables are already in the model.

The final linear model predicting 4-month weight loss, summarized in Table 4, was generated with baseline variables, the *app-use* component, the *app-calories* component, and the frequency of self-weighing at 8 weeks. The model revealed that the components of *app-use* and *app-calories* as well as the frequency of weighing were significant at the *P*=.01 level. After accounting for these variables, treatment assignment and age were found to be not significant. The adjusted *R*^2^ value for the model was 0.32. If the *app-exercise* component (PC_3_) was included, it was found to be not significant.

**Table 4:** Linear regression model summary predicting 4-month weight loss with 8-week data.

Coefficients	b (SE)	*t* test (*df*)	*P* value
Intercept	−1.89E−02 (1.67E−02)	−1.14 (148)	.25
App-use (PC_1_^a^)	4.51E−03 (1.35E−03)	3.34 (148)	.001
App-calories (PC_2_^b^)	6.20E−03 (2.06E−03)	3.01 (148)	.003
Treatment assignment	8.71E−03 (7.30E−03)	1.19 (148)	.23
Age	3.98E−04 (3.97E−04)	1 (148)	.32
Self-weighing frequency at 8 weeks	6.58E−04 (2.41E−04)	2.73 (148)	.007

^a^PC_1_: principal component 1.

^b^PC_2_: principal component 2.

For 12-month predictions (Table 5), the components of *app-use* and *app-calories* were not statistically significant predictors. Treatment assignment and the component of *app-exercise* were also not significant. However, age and the frequency of self-weighing were significant predictors.

**Table 5:** Linear regression results predicting 12-month weight loss with 8-week data.

Coefficients	b (SE)	*t* test (*df*)	*P* value
Intercept	−6.69E−02 (2.62E−01)	−2.55 (147)	.01
App-use (PC_1_^a^)	2.96E−03 (2.05E−03)	1.45 (147)	.15
App-calories (PC_2_^b^)	4.01E−03 (3.17E−03)	1.26 (147)	.21
Treatment assignment	6.27E−03 (1.12E−02)	0.56 (147)	.58
Age	1.34E−03 (6.08E−04)	2.21 (147)	.03
Self-weighing frequency at 8 weeks	7.84E−04 (3.77E−04)	2.08 (147)	.04

^a^PC_1_: principal component 1.

^b^PC_2_: principal component 2.

Although predicting 12-month weight loss with baseline and electronic data from the first 8 weeks resulted in low accuracy, using only 4-month weight loss as a predictor resulted in an *R*^2^ of 0.31 (ρ=.52; multi-class AUC=.66).

## Discussion

### Principal Findings

Our results suggest that early study self-monitoring data, specifically PCs representing app use and self-monitoring of caloric intake and frequency of self-weighing, predict weight loss at 4 months, consistent with previous research indicating early self-monitoring predictors of treatment success [17,19]. Data from the first 8 weeks generated slightly more accurate weight loss predictions than data from the first 4 weeks; these results are consistent with those of Unick et al [20], who found that weight loss at 1 and 2 months was associated with 8-year weight loss. Predicting 12-month weight loss using early study data proved to be more challenging; however, 4-month weight loss was predictive of 12-month weight loss, which follows the results of previous research that indicated that early weight change can predict long-term weight change [18,20,49].

Our mediation results (Table 2) showed that *app-use* and the frequency of self-weighing partially mediated the relationship between treatment assignment and weight loss during the first 4 months. This suggests that the intervention not only directly affected 4-month weight loss but also indirectly affected weight change through weighing and app use behavior. However, the intervention mainly had a short-term effect because the results demonstrate a full mediation effect on the association between treatment assignment and 12-month weight loss through the 4-month weight loss.

Predictive modeling results revealed consistent self-monitoring to be an important aspect of 4-month weight loss, which reflects the findings of many previous studies [1,2]. The decrease in app use over time also followed a similar pattern to previous findings in web-based and traditional self-monitoring studies [19,50,51]. Our descriptive results on the differences in logging trends between the treatment groups provide preliminary evidence that self-monitoring with regular feedback may improve self-monitoring consistency, which differs from the results of some previous studies that show that there is no difference in adherence between a treatment group that receives feedback and a group that receives no feedback [3].

The regression results also demonstrated that the PC representing exercise self-monitoring was not a significant predictor of short-term weight loss. Previous research has shown that exercise is more crucial in maintaining weight than losing weight [52-54]. Although we did not measure exercise (ie, the behavior) in the study, self-monitoring exercise seemed to add little benefit to short-term weight loss. Nonetheless, weight loss interventions should continue to encourage participants to increase their physical activity because of its role in weight maintenance; however, interventionists could focus on stressing the importance of self-monitoring caloric intake and self-weighing over monitoring exercise during the early intense weight loss period.

Overall, our results indicate that short-term weight loss leads to long-term weight loss, which is consistent with previous research. It is likely that the intervention establishes certain behaviors that induce weight loss at 4 months, some of which are continued at 12 months. The counselor-initiated condition seemed to establish more of these behaviors by 4 months, and the counselor-initiated–treatment participants were more successful at self-monitoring.

### Strengths and Limitations

The strengths of the study include that it was a randomized clinical trial and one of the few studies that used data from a popular commercial app. In addition, the context of the randomized intervention study allowed us to conduct a causal mediation analysis to understand whether the intervention assignment directly and indirectly affected weight loss. Further, this is one of few weight control studies in the military.

By including a behavioral run-in period of dietary self-monitoring (as in previous studies [17]) in addition to requiring a medical clearance letter, it is possible that the participants may have been more motivated and well-informed about the study activities than those who did not complete these tasks. However, an examination of the characteristics of the randomized individuals compared with those of the individuals who were not randomized [27] showed that higher educational status was the only independent predictor of randomization. It is also interesting to note that it was initially expected that most of the participants would be motivated to join the study to assist them in passing the military fitness test. However, when the motivators for weight loss in this sample were examined, it was found that the most frequently endorsed motivators were improved physical health, improved fitness, improved quality of life, and a desire for longevity [55].

A limitation of using a commercial app in analysis is that the researchers have little control over the format in which they collect and receive data. In this study, a large portion of the nutrient information was not available because of the methods that the participants chose to log their calories. For instance, some participants logged calories directly without food description or nutrient details (eg, a lump sum of 2000 calories for the full day without further details); therefore, we were unable to use the nutrient reports in the analysis. We were also unable to include data from 57 participants because of technical errors in the data retrieval process because of a clerical mistake. This differential missingness between the 2 intervention conditions could have introduced bias; however, we note that the cause of the missing data was the data acquisition process and unrelated to the identity or behavior of the participants.

### Conclusions

We found that long-term weight loss was completely mediated through short-term weight loss, reiterating the importance of interventions that produce strong successes quickly. Approximately one-third of the 12-month weight loss was explained by weight loss at 4 months. More than half of the effect of the behavioral intervention on weight loss at 4 months was mediated through self-monitoring app use and self-weighing frequency, indicating the potency of these self-regulatory behaviors. As we did not find evidence of an effect of self-monitored exercise, it suggests that diet self-monitoring should be prioritized for successful weight loss.

## Appendix

Multimedia Appendix 1 Supplemental analysis tables.

## Abbreviations

ACME: average causal mediation effect
AHEAD: Action for Health in Diabetes
AUC: area under the receiver operating characteristic curve
PC: principal component
PCA: principal component analysis
TE: total effect
