# Supplementary material for: Assessing the Contribution of Self-Monitoring Through a Commercial Weight Loss App: Mediation and Predictive Modeling Study
Source: JMIR Mhealth Uhealth. 2021 Jul 14;9(7):e18741. doi: 10.2196/18741 (PMC8319781; doi:10.2196/18741)
Supplement: Multimedia Appendix 1 [file mhealth_v9i7e18741_app1.docx]

# Multimedia Appendix – Supplemental Analysis Tables

This is a Multimedia Appendix to a full manuscript published in JMIR mHealth and uHealth.

Farage G, Simmons C, Kocak M, Klesges RC, Talcott GW, Richey P, Hare M, Johnson KC, Sen S, Krukowski R

Assessing the contribution of self-monitoring through a commercial weight loss app: mediation and predictive modeling

JMIR Mhealth Uhealth 2021;0(0):e0

URL: https://mhealth.jmir.org/2021/0/e0/

doi: 10.2196/18741

Table S1. Linear regression results of 4-month weight loss on the treatment assignment

|  | *β* | SE | *t(df)* | *P* |
| --- | --- | --- | --- | --- |
| Intercept | -1.49E-02 | 1.54 E-02 | -0.967(151) | 0.33 |
| Treatment assignment | 3.42E-02 | 6.59E-03 | 5.16(151) | <0.0001 |
| Age | 6.69E-04 | 4.35E-04 | 1.54(151) | 0.126 |

Table S2. Linear regression results of the App-use component (PC_1_) on the treatment assignment

|  | *β* | SE | *t(df)* | *P* |
| --- | --- | --- | --- | --- |
| Intercept | -2.62 | 8.24 E-01 | -3.18(151) | 0.0018 |
| Treatment assignment | 3.21 | 3.51E-01 | 9.13(151) | <0.0001 |
| Age | 3.77E-02 | 2.32E-02 | 1.63(151) | 0.106 |

Table S3. Linear regression results of self-weighing frequency on the treatment assignment

|  | *β* | SE | *t(df)* | *P* |
| --- | --- | --- | --- | --- |
| Intercept | 46.0 | 11.1 | 4.12(151) | <0.0001 |
| Treatment assignment | 35.6 | 4.75 | 7.50(151) | <0.0001 |
| Age | 0.315 | 0.313 | 1.00(151) | 0.317 |

Table S4. Linear regression results of 12-month weight loss on the treatment assignment

|  | *β* | SE | *t(df)* | *P* |
| --- | --- | --- | --- | --- |
| Intercept | -4.17E-02 | 2.45E-02 | -1.01(127) | 0.0911 |
| Treatment assignment | 3.15E-02 | 1.02E-02 | 3.09(127) | 0.0024 |
| Age | 1.27E-02 | 6.83E-04 | 1.86(127) | 0.0652 |

Table S5. Linear regression results of 12-month weight loss on 4 month-weight loss

|  | *β* | SE | *t(df)* | *P* |
| --- | --- | --- | --- | --- |
| Intercept | -4.07E-03 | 5.39E-03 | -0.76(127) | 0.45 |
| 4-Month weight loss | 7.82E-01 | 9.85E-02 | 7.94(127) | <0.0001 |

Table S6. Accuracy of 4-week models

| Model | 4-Month Prediction | | | 12-Month Prediction | | | |
| --- | --- | --- | --- | --- | --- | --- | --- |
|  | R^2^ | *ρ* | mAUC | R^2^ | ρ | mAUC | |
| Baseline variables (i.e., age and treatment assignment) | .16 | .41 | .65 | .06 | .25 | | .58 |
| App-use (PC_1_) + App-calories (PC_2_) + Self-weighing frequency | .24 | .50 | .69 | .04 | .24 | | .55 |
| Baseline. + App-use (PC_1_) +  App-calories (PC_2_) + Self-weighing frequency | .26 | .53 | .71 | .06 | .27 | | .59 |

Table S7. 4-month and 12-month mediation sensitivity analysis results. ${\tilde{\boldsymbol{R}}}_{\boldsymbol{M}}^{\boldsymbol{2}}$ and ${\tilde{\boldsymbol{R}}}_{\boldsymbol{Y}}^{\boldsymbol{2}}$ represents respectively the proportions of the total variance in the mediator and the outcome variables, which would be explained by an unobserved pretreatment confounder [43].

|  | $\tilde{R}_{M}^{2}\tilde{R}_{Y}^{2}$ |
| --- | --- |
|  | *4-MONTH MEDIATION* |
| App-use (PC_1_) | *.*051 |
| Self-weighing frequency | *.*072 |
|  | *12-MONTH MEDIATION* |
| 4-Month Weight Loss | *.*14 |
